# At Home at the Villa Imperiale

**Published:** 1890-10

**Authors:** 


### AT HOME AT THE VILLA IMPERIALE.

### A DAY WITH THE PRINCE OF WALES.

During his stay at Homburg the Prince of Wales is living, as usual, at the Villa Impériale. His Royal Highness's life is most regular. About seven o'clock in the morning he goes to the spring, which is a few minutes' walk from the house, where he finds the Duke of Cambridge and the Duke of Teck, besides a curious crowd. He drinks two or three glasses of water, and then walks up and down among the many guests, listening to the band. At nine o'clock he returns to the house and breakfasts on the veranda, after which he reads the newspapers until ten. Lying on the chair beside him are always to be seen numerous pamphlets, some French works on strategy, and a huge pile of Blue-books. From ten until one the Prince works. At 1 o'clock he lunches, usually at the Park Hotel, but sometimes, though less often, on the terrace of the Kurhaus. Afterwards he drives in the mountains, or has tea on the balcony. At 7 he dines with about half-a-dozen guests, on the terrace of the Kurhaus, while the band plays in the Kurgarten Pavilion. After dinner, about nine o'clock, the Prince and his guests go down to the Kurgarten to listen to the concert, sometimes seated in one of the first rows of chairs, sometimes walking up and down. At eleven the Prince returns home. He seldom goes to bed later than midnight. He looks exceedingly well, and every one is enchanted with his simplicity and kindness. Before going to bed the Prince takes one or two glasses of Apollinaris water with lemon juice.

